# Kaniadakis Functions beyond Statistical Mechanics: Weakest-Link Scaling, Power-Law Tails, and Modified Lognormal Distribution

**DOI:** 10.3390/e24101362

**Published:** 2022-09-26

**Authors:** Dionissios T. Hristopulos, Anastassia Baxevani

**Affiliations:** 1 School of Electrical and Computer Engineering, Technical University of Crete, 73100 Chania, Greece; 2 Department of Mathematics and Statistics, University of Cyprus, Nicosia 1678, Cyprus

**Keywords:** Kaniadakis exponential, modified lognormal distribution, earthquake recurrence times, Weibull distribution, power-law tail, precipitation, flow in random media, tensile strength, 02.50.Fz, 02.60.Ed, 89.60.-k, 92.60.Ry, 05.10.Ln, 60G15, 60G60, 62F40, 62H11, 62G05, 65C05

## Abstract

Probabilistic models with flexible tail behavior have important applications in engineering and earth science. We introduce a nonlinear normalizing transformation and its inverse based on the deformed lognormal and exponential functions proposed by Kaniadakis. The deformed exponential transform can be used to generate skewed data from normal variates. We apply this transform to a censored autoregressive model for the generation of precipitation time series. We also highlight the connection between the heavy-tailed κ-Weibull distribution and weakest-link scaling theory, which makes the κ-Weibull suitable for modeling the mechanical strength distribution of materials. Finally, we introduce the κ-lognormal probability distribution and calculate the generalized (power) mean of κ-lognormal variables. The κ-lognormal distribution is a suitable candidate for the permeability of random porous media. In summary, the κ-deformations allow for the modification of tails of classical distribution models (e.g., Weibull, lognormal), thus enabling new directions of research in the analysis of spatiotemporal data with skewed distributions.

## 1. Introduction

Several physical processes exhibit asymmetric probability distributions which deviate from the Gaussian law (e.g., the exponential, gamma, Weibull, lognormal, Pareto, and generalized Pareto models) [1,2,3,4,5,6,7,8]. Skewed probability distributions describe various geophysical variables, including the amount and duration of precipitation over a certain time window [9,10,11,12], the waiting times (recurrence or interevent times) between consecutive earthquakes occurring over a given area [6,7,8], the fluid permeability of geological porous media [13,14,15,16], as well as the mechanical strength distribution of the earth’s crust [17,18] and various technological brittle materials [19,20,21,22].

A feature of particular interest is the behavior of the tail(s) of a probability distribution, because the tails define the probabilities of extreme events. Distributions are characterized as sub-exponential (if their tail decays slower than the exponential) and super-exponential in the opposite case. The same function (e.g., the Weibull model) may exhibit transitions from sub-exponential to super-exponential by changing the value of a key parameter; in the Weibull case this is the modulus *m*: m<1 leads to a sub-exponential and m>1 to a super-exponential tail. Sub-exponential models are called heavy-tailed if the asymptotic behavior of the probability for large events decays algebraically, i.e., $P\left(X>x\right)∼x^{-\alpha }$, where α>0.

In particular, probability distributions with power-law tails are ubiquitous in natural phenomena [23,24]. Such power laws can be generated by means of different physical mechanisms as described in ([23], Chap. 14). Multiple mechanisms including (but not limited to) phase transitions, self-organized criticality, optimization, multiplicative processes, and interdependence in complex systems [25,26,27,28,29] lead to power laws. This fact explains, to some extent, the omnipresence of power laws in physics, biology, earth science, cosmology, ecology, finance, and other disciplines. In addition, the detection of power-law distributions in data has significant impact on statistical analysis and forecasting [30].

Approximately twenty years ago, Kaniadakis introduced the κ-deformed exponential and κ-deformed logarithmic functions [31,32,33,34]. These functions provide a springboard for the construction of generalized, flexible probability distributions whose tails are controlled by the deformation parameter κ. Based on the deformed functions, extensions of the known generalized gamma, Weibull, generalized logistic, and exponential models can be constructed which exhibit power-law tails; explicit expressions for the probability functions of these models are given in [35]. The Kaniadakis functions have found applications in plasma physics [36,37], gravitational systems [38], income analysis [39,40,41], epidemiology [42], and other fields (see [35] for a more comprehensive list of applications). In particular, κ-statistical theory uses the κ-exponential function to generalize the Maxwell–Boltzmann distribution, leading to distributions with power-law tails. Distribution models with flexible tails are also needed in earth science, where datasets often exhibit tail behavior that is not adequately captured by classical distribution models [43,44,45].

The motivation for this work is the need for flexible statistical models that can adapt to the diversity of earth science data and also provide physical intuition. Our objective is to explore the possibilities created by the parameter κ which appears in the deformed exponential and logarithmic functions. This investigation leads to novel applications of the Kaniadakis functions in geostatistics, material fracture, precipitation, and fluid flow modeling.

The main contributions of this paper are as follows.

- We show (Section 3) that the κ-deformed exponential and logarithmic functions (henceforth, κ-exponential and κ-logarithm) can be used to define normalizing transforms for non-Gaussian data, which extend the well-known (in statistics) Box–Cox family of transformations [46].
- We formulate an autoregressive, intermittent precipitation model based on the κ-modified Box–Cox transform in Section 3.3. We show that the resulting precipitation time series has higher “peaks” than those obtained with the Box–Cox transform with the same parameter value.
- We review the κ-Weibull distribution focusing on its connection with weakest-link theory (Section 4). This demonstrates that the κ-Weibull is a physically motivated generalization of the classical Weibull distribution for the mechanical strength of brittle materials, unlike modified Weibull distributions which fail to satisfy the weakest-link principle.
- We show that for several physical quantities, including the thickness of magmatic sheet intrusions, the tensile strength of steel, earthquake waiting times, and precipitation amounts the κ-Weibull distribution provides a better fit than the Weibull according to model selection criteria.
- We introduce the κ-lognormal distribution, which provides a deformation of the lognormal with lighter tails than the latter in Section 5. The κ-lognormal can be used to model asymmetric data distributions which concentrate more probability mass around the median than the lognormal. We discuss the importance of the generalized mean (power mean) of the lognormal distribution for estimating the effective permeability of heterogeneous porous media, and we calculate the generalized mean of the κ-lognormal distribution.

## 2. Mathematical Preliminaries

Let (Ω,F,P), where Ω is the sample space, F is a σ-field of subspaces of Ω, and *P* is a probability measure, define a probability space. A real-valued, scalar random variable X(ω) is defined by the mapping X:Ω→R, where R is the set of real numbers [47]. Furthermore, a stochastic process X(t;ω) indexed by the time t∈R is defined by the mapping X:R×Ω→R. In the following, the dependence on the state index ω is suppressed for convenience. In addition, the random variable *X* represents a load or the waiting time between consecutive “failure” events (e.g., earthquakes), or some other asymmetrically distributed variable.

The function F(x):P(X≤x):R→[0,1] defines the cumulative distribution function (CDF) of *X*, or the marginal CDF of a stationary stochastic process {X(t)}. The expectation of *X*, assuming it is mathematically well defined, is given by means of E[X]=∫dF(x)x.

Assuming that F(x) is at least once differentiable, the probability density function (PDF) is given by the first derivative of the CDF, i.e., f(x)=dF(x)/dx.

The CDF is related to the so-called survival function, also known as the reliability function, which is given by S(x)=1−F(x). Whereas F(x) is a monotonically increasing function, S(x) is monotonically decreasing. The term “survival function” comes from reliability engineering: if *X* represents the strength or critical loading of a given system, F(x) is the probability that the system fails at loading level X≤x; then, S(x) is respectively the probability that the system remains intact (survives) at this loading level.

The quantile function, Q(p), where p∈[0,1], returns the value $x_{p}\in R$ such that $F(x_{p})=p$. Hence, Q(·) is the inverse of the CDF.

The hazard rate, also known as the hazard function, h(x) represents the conditional probability that the system fails for X∈(x,x+δx] where δx≪1, conditioned on the survival of the system for X≤x. Let A denote the event that the system survives at level *x* and B denote the event of system failure in the interval [x,x+δx]. Then, by using the definition of conditional probabilities, h(x)=P(B|A)=P(B∩A)/P(A), the hazard rate is given by the following ratio:

(1) $$h\left(x\right)=\lim_{\delta x\to 0}\frac{P\left(x<X\leq x+\delta x\right)}{S(x)\;\delta x}=\frac{f(x)\delta x}{S(x)\delta x}=\frac{f(x)}{S(x)}.$$

The asymptotic behavior of the hazard rate determines the probability of system failure with increasing load. The hazard rate for x→∞ is determined by the tail of the probability functions f(x) and S(x). For certain probability models, e.g., the exponential and the gamma, h(x) tends to a constant as x→0; for the Weibull model with modulus m>1, h(x) tends to zero, whereas for models with power-law tails, the lognormal, and the Weibull model with m<1, h(x) diverges as x→∞. The hazard rate is an important factor in seismic risk assessment [48].

### 2.1. The κ-Exponential Function

The κ-generalized exponential is a one-parameter generalization of the exponential function, proposed by Kaniadakis [31,34]:

(2) $$\exp_{\kappa }\left(x\right)={\left(\sqrt{1+x^{2}\kappa^{2}}+x\kappa \right)}^{1/\kappa },$$

with 0≤κ<1 and x∈R. The first few terms of the Taylor expansion of $\exp_{\kappa }\left(x\right)$, reported in [49], are given by

(3) $$\exp_{\kappa }\left(x\right)=1+x+\frac{x^{2}}{2}+\left(1-\kappa^{2}\right)\frac{x^{3}}{3!}+\left(1-4\kappa^{2}\right)\frac{x^{4}}{4!}+\ldots \;.$$

The emerging pattern persists for higher orders, i.e., terms of $O(x^{3})$ consist of the ordinary exponential expansion and a κ-dependent correction. The κ-exponential is expressed as the following power series [49]:

(4) $$\exp_{\kappa }\left(x\right)=\sum_{n=0}^{\infty }\xi_{n}\left(\kappa \right)\frac{x^{n}}{n!}.$$

The functions ${\left\{\xi_{n}\left(\kappa \right)\right\}}_{n=0}^{\infty }$ are polynomials of κ defined by the recurrence relations

(5) $$\begin{array}{cc} \; & \xi_{0}\left(\kappa \right)=\xi_{1}\left(\kappa \right)=1,\;n=0,1, \end{array}$$

(6) $$\begin{array}{cc} \; & \xi_{n}\left(\kappa \right)=\prod_{j=1}^{n-1}\left[1-(2j-n)\kappa \right]=\xi_{n-2}\left(\kappa \right)\left[1-{\left(n-2\right)}^{2}\kappa^{2}\right],\;n>1. \end{array}$$

The polynomials $\xi_{n}\left(\kappa \right)$ for the first seven orders are given by

(7) $$\begin{array}{cc} \; & \xi_{0}\left(\kappa \right)=\xi_{1}\left(\kappa \right)=\xi_{2}\left(\kappa \right)=1, \end{array}$$

(8) $$\begin{array}{cc} \; & \xi_{3}\left(\kappa \right)=1-\kappa^{2}, \end{array}$$

(9) $$\begin{array}{cc} \; & \xi_{4}\left(\kappa \right)=1-4\kappa^{2}, \end{array}$$

(10) $$\begin{array}{cc} \; & \xi_{5}\left(\kappa \right)=\left(1-\kappa^{2}\right)\left(1-9\kappa^{2}\right), \end{array}$$

(11) $$\begin{array}{cc} \; & \xi_{6}\left(\kappa \right)=\left(1-4\kappa^{2}\right)\left(1-16\kappa^{2}\right). \end{array}$$

It follows from Equation (3) that when x→0 or κ→0, $\exp_{\kappa }\left(x\right)$ converges to the ordinary exponential, i.e.,

(12) expκ(x)x→0∼exp(x),

(13) expκ(x)κ→0∼exp(x).

Equation (2) shows that the asymptotic behavior of $\exp_{\kappa }\left(x\right)$ as x→±∞ follows a power law [34,49], i.e.,

(14) expκ(x)x→±∞∼|2κx|±1/κ.

Based on the above, for x→+∞ the modified exponential exhibits a heavy tail, i.e. $\exp_{\kappa }\left(-x\right)∼{\left(2\kappa x\right)}^{\;-1/\kappa }$. Hence, $\exp_{\kappa }\left(x\right)$ can be used to model subexponential probability distributions which are suitable for heavy-tailed data.

The κ-exponential can also be introduced as the solution of a linear, first-order ordinary differential equation (ODE) with time-dependent rate [42]. Consider the ODE

(15) $$\frac{df(x)}{dx}=-r\left(x\right)\;f\left(x\right),\;\mathrm{with}\;\mathrm{initial}\;\mathrm{condition}\;f\left(0\right)=1,$$

where r(x) is the following *x*-dependent rate function

(16) $$r\left(x\right)=\frac{\beta }{\sqrt{1+\kappa^{2}\beta^{2}x^{2}}}.$$

The solution of the ODE (15) is given by the function $f\left(x\right)=\exp_{\kappa }\left(-\beta x\right)$. In case κ=0, then r(x)=β, and the rate equation is solved by the standard exponential function f(x)=exp(−βx).

### 2.2. The κ-Logarithm Function

The inverse of the κ-exponential is the κ-logarithm, defined by the following function for x>0:

(17) $$\ln_{\kappa }\left(x\right)=\frac{x^{\kappa }-x^{-\kappa }}{2\kappa }.$$

The κ-logarithm satisfies the equation $\ln_{\kappa }\exp_{\kappa }\left(x\right)=x$. In addition, it respects the κ-symmetry property $\ln_{\kappa }\left(x\right)=\ln_{-\kappa }\left(x\right)$. A Taylor expansion of $x^{\kappa }$ and $x^{-\kappa }$ around κ=0 leads to

(18) $$\lim_{\kappa \to 0}\ln_{\kappa }\left(x\right)=\ln x\;.$$

The first and second derivatives of the κ-logarithm are respectively given by

(19a) $$\frac{d\ln_{\kappa }\left(x\right)}{dx}=\frac{1}{2}\left(x^{\kappa -1}+x^{-\kappa -1}\right),$$

(19b) $$\frac{d^{2}\ln_{\kappa }\left(x\right)}{dx^{2}}=\frac{\kappa -1}{2}\;x^{\kappa -2}-\frac{\kappa +1}{2}\;x^{-\kappa -2}\;.$$

Based on Equation (19a), the first derivative of the κ-logarithm is positive; therefore $\ln_{\kappa }\left(x\right)$ is a monotonically increasing function. Based on Equation (19b), the second derivative of $\ln_{\kappa }\left(x\right)$ is negative for 0<κ<1; therefore, $\ln_{\kappa }\left(x\right)$ is a concave function for κ∈(0,1).

## 3. Nonlinear Transformation of Data Based on the κ-Logarithm

Nonlinear, monotonic transformations are often applied to non-Gaussian data in order to restore normality [50,51,52]. This procedure, known as Gaussian anamorphosis, enables the use of data processing methods that are based on Gaussian assumptions. Various transforms are used in practice, including the Box–Cox [46] and Yeo–Johnson [53]. Such transforms can be generalized by means of the Kaniadakis functions. Below we focus on Box–Cox but the same arguments can be used for other transforms.

### 3.1. Box–Cox Transform and the Replica Trick

A widely used normalizing transformation in statistics is the so-called Box–Cox transform (BCT) [46]; the one-parameter version of BCT is given by the monotonic function

(20) $$g_{BC}\left(x\right)=\frac{x^{\lambda }-1}{\lambda },\;where\;\lambda \in R,\;x>0.$$

The BCT is applied to skewed (non-Gaussian) data so that the transformed variable $y=g_{BC}\left(x\right)$ is better approximated by the Gaussian distribution. The BCT is applied to both time series and spatial data [52].

It is interesting to note that if λ<0, $g_{BC}\left(x\right)$ takes positive (negative) values for x<1 (x>1), whereas if λ>0, the $g_{BC}\left(x\right)$ takes positive (negative) values for x>1 (x<1). The BCT value for λ=0 can be obtained by using either l’Hopital’s rule or the Taylor expansion. The Taylor expansion of $x^{\lambda }$ around λ=0 shows that $x^{\lambda }=1+\lambda \ln x+O\left(\lambda^{2}\right)$. Therefore,

(21) $$\lim_{\lambda \to 0}\frac{x^{\lambda }-1}{\lambda }=\ln x\;.$$

Equation (21) shows that the logarithmic transform is a special case of the BCT for λ=0. The inverse BCT is given by $h_{BC}\left(y\right)≜g_{BC}^{-1}\left(y\right)={\left(\lambda y+1\right)}^{1/\lambda }$.

In a different context, Edwards and Anderson [54] introduced the famous replica trick, which is also based on Equation (21), to study spin glasses. The replica trick is used to calculate the ensemble average (over the magnetic disorder) of the logarithm of the spin glass partition function, i.e., $\bar{\ln Z}$. By using the replica trick, $\bar{\ln Z}$ is calculated by first evaluating $\bar{g_{BC}\left(Z\right)}$, with λ=n∈N denoting the number of replicas (identical copies of the system), and then taking the limit n→0.

### 3.2. The κ-Logarithmic Transform

The κ-logarithm transform (KLT) is a nonlinear, monotonic transformation from $R_{+}\to R$. Therefore, it can be used like the BCT for the Gaussian anamorphosis of positive-valued data. The KLT takes the form

(22) $$g_{KL}\left(x\right)=\ln_{\kappa }\left(x\right)=\frac{x^{\kappa }-x^{-\kappa }}{2\kappa },\;\kappa \in R,\;x>0.$$

**Figure 1:**
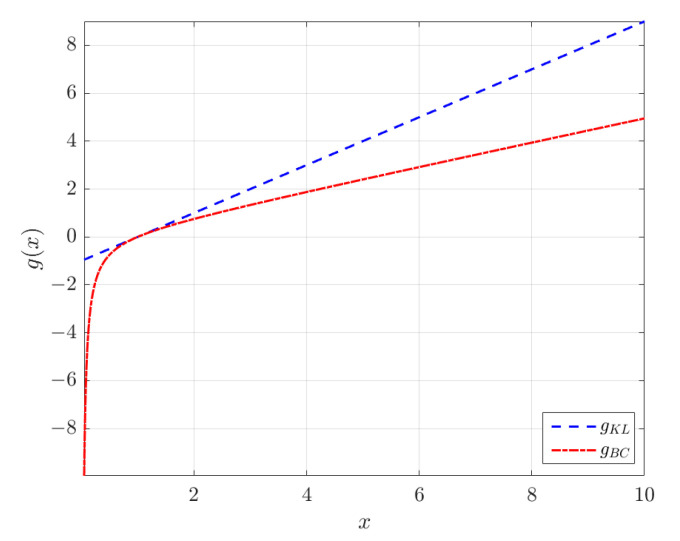
Plots of the Box–Cox and κ-logarithmic transform for λ=κ=1 (λ is the Box–Cox parameter and κ is the deformation parameter of the Kaniadakis logarithm).

**Figure 2:**
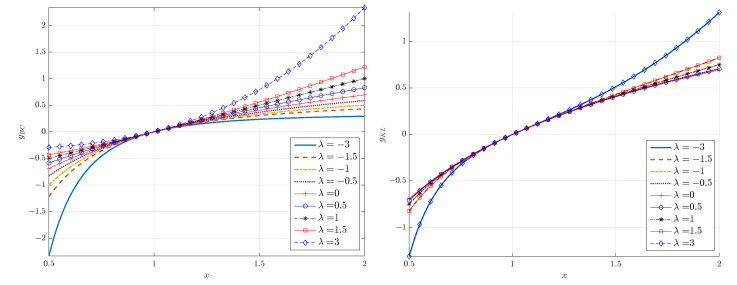
Plots of the Box–Cox (**left**) and κ-logarithmic (**right**) transform for different values of λ=κ (λ is the Box–Cox parameter and κ is the deformation parameter of the Kaniadakis logarithm).

Equation (18) shows that the logarithmic transform is a special case of the KLT for κ→0, as it is a special case of the BCT. To understand how the transformation works, consider the special cases κ=λ=1 shown in Figure 1. The choice λ=1 for the BCT is a simple linear shift of *x* to x−1, whereas setting κ=1 in the KLT leads to the nonlinear transformation $\frac{x^{2}-1}{2x}$. The latter tends to the linear transformation x/2 for x≫1. Figure 2 shows the two transformations for different values of λ=κ. Notice that the KL transformation is symmetric with respect to κ↦−κ. The inverse of the KLT is given by $h_{KL}\left(y\right)=\exp_{\kappa }\left(y\right)$.

### 3.3. Application to Precipitation Modeling

Autoregressive (AR) models are used for different meteorological processes which exhibit memory. AR models exhibit short-term memory: the present depends on the past via the *p* most recent values of the process

(23) $$y_{t}=m+\sum_{i=1}^{p}\phi_{i}\;\left(y_{t-i}-m\right)+\sigma_{\epsilon }\;\epsilon_{t},$$

where $m=E[y_{t}]$ is the expectation of $y_{t}$, the set ${\left\{\phi_{i}\right\}}_{i=1}^{p}$ comprises the real-valued auto-regressive coefficients, $\left\{\epsilon_{t}\right\}$ is the innovation process, and $\sigma_{\epsilon }$ is its standard deviation. The innovation is typically considered to be standard Gaussian white noise, i.e., $\epsilon_{t}∼\left(0,1\right)$, leading to a normally distributed time series ${\left\{y_{t}\right\}}_{t=1}^{T}$.

Daily precipitation usually displays intermittency (i.e., intervals of zero precipitation) in addition to temporal variability of its intensity. These features can be modeled by using a censored autoregressive time series model [55]. Then, the amount of daily precipitation is given by $x_{t}=y_{t}\;\theta \left(y_{t}-y_{c}\right)$, where θ(·) is the unit step function and $y_{c}$ is a censoring threshold. The threshold is selected so that $F_{y}\left(y_{c}\right)=p_{0}$, where $p_{0}$ is the probability of observing a dry day. The censored AR model leads to a truncated normal distribution for $x_{t}$. Because this distribution does not adequately reflect the extreme values of precipitation, in practice the daily precipitation is given by means of the following censored and transformed autoregressive process,

(24) $$x_{t}=h\left[\;y_{t}\;\theta \left(y_{t}-y_{c}\right)\right]-h\left(0\right),$$

where h(·):R→R is a monotonically increasing transform. The application of h(·) introduces skewness and increases the weight in the right tail of the distribution. The subtracted term h(0) restores the zero precipitation values after application of the transform. The function h(·) could, for example, represent the inverse BC or KL transforms, i.e., $h\left(y\right)={\left(\lambda y+1\right)}^{1/\lambda }$ or $h\left(y\right)=\exp_{\kappa }\left(y\right)$, respectively.

**Figure 3:**
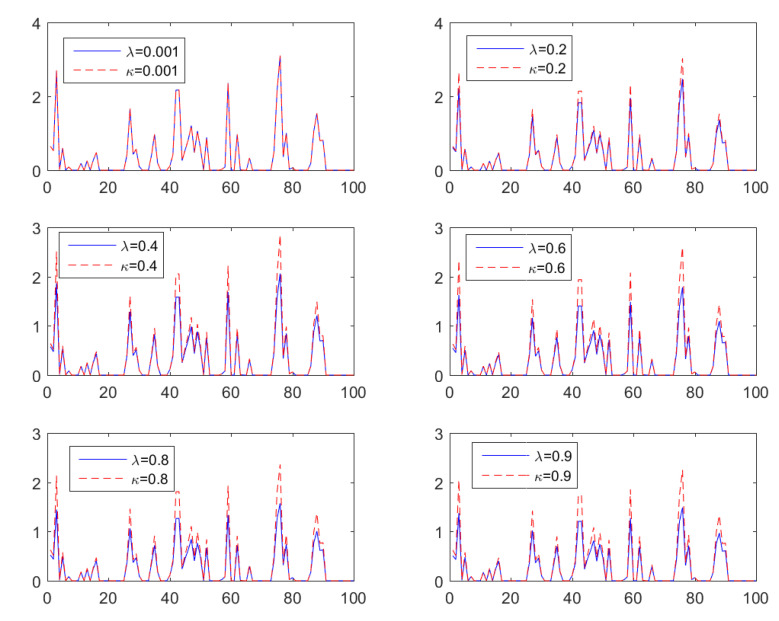
Realizations of six time series generated by the censored and transformed AR(1) model of Equation (24) with $\phi_{1}=0.5$ and $\sigma_{\epsilon }=0.6$. The nonlinear transform uses BCT (blue, continuous lines) and KLT (red, broken lines) for κ=λ∈{0.001,0.2,0.4,0.6,0.8,0.95}.

Figure 3 presents six realizations of the censored AR(1) model of Equation (24) using both the inverse BCT and KLT as the nonlinear transformation h(·), with equal values of κ and λ in each frame. The AR(1) model of Equation (24) is applied with $\phi_{1}=0.5$ and $\sigma_{\epsilon }=0.6$. The time series exhibit intermittent behavior due to censoring. The difference between BCT and KLT is negligible for small values of κ=λ. For κ≈0, both transforms yield a censored lognormal process, because $h_{KL}\left(y\right)$ and $h_{BC}\left(y\right)$ converge to the normal exponential for κ=λ→0. The KLT peaks become progressively higher compared to the BCT peaks as κ (and λ) increase. This behavior is due to the following inequality between the inverse transforms

$$h_{KL}\left(y\right)={\left(\sqrt{1+\kappa^{2}\;y^{2}}+\kappa y\right)}^{1/\kappa }>h_{BC}\left(y\right)={\left(1+\kappa y\right)}^{1/\kappa },\;\kappa >0.$$

The increase in the relative height of KLT-based versus BCT-based peaks with increasing κ should not be confused with the fact that the peaks of $x_{t}$ are highest for κ=λ=0, i.e., when h(·) is the exponential function (herein κ=0 implies the limit κ→0). This behavior is due to the inequality $\exp_{\kappa }\left(y\right)<\exp\left(y\right)$ for all y≥0 and for 0<κ≤1, e.g., [49].

## 4. The κ-Weibull Distribution and Its Applications

Complex systems involve collections of interacting units and often exhibit probability distributions with power-law tails [23]. A long (power-law) right tail of the PDF implies that the occurrence probability for extreme events decays slowly.

**Figure 4:**
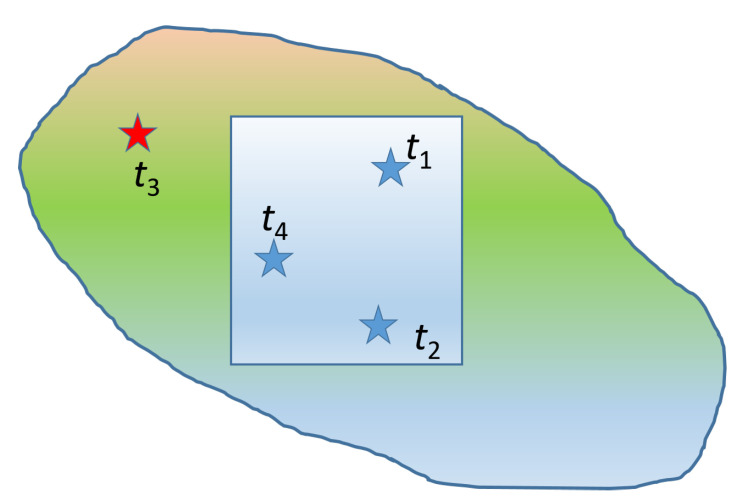
Schematic illustrating how long tails can emerge if the observation window (blue square) is a nested insider a larger, interacting system (see text for explanation). Blue stars indicate events inside the observation window, while the red star refers to an event outside the window.

One mechanism that can lead to the emergence of long tails is due to limited size of the observation window as illustrated in Figure 4. Consider an observation domain (indicated by the square domain in the center) which is part of a larger interconnected system (denoted by the oval-shaped area). Let us assume that in the entire system, the failure events occur at times $t_{1}<t_{2}<t_{3}<t_{4}$. Furthermore, assume that the interevent times $t_{2}-t_{1}$, $t_{3}-t_{2}$, and $t_{4}-t_{3}$ follow a distribution which does not necessarily have a heavy tail. However, since the observed system involves only the square domain, the observed interevent times are $t_{2}-t_{1}$ and $t_{4}-t_{2}$; the latter results by adding the true interevent times $t_{3}-t_{2}$ and $t_{4}-t_{3}$, leading to a larger period of quiescence than if the entire system were taken into account. The repeated occurrence of events outside the observed domain can thus inflate waiting times and transfer probability weight from the low and middle range of the probability distribution to the right tail.

In such cases, the classical Weibull distribution may not be adequate because it has a right tail which decays at best (i.e., for m<1) as a stretched exponential. In contrast, the κ-Weibull distribution has a flexible power-law tail with exponent equal to −m/κ.

### 4.1. κ-Weibull Probability Functions

The Weibull distribution is most simply defined in terms of its survival function $S\left(x\right)=\exp\left[-{\left(x/x_{s}\right)}^{m}\right]$, for x≥0, where $x_{s}$>0 is the scale parameter and *m*>0 is the shape parameter or Weibull modulus. The Weibull distribution is used in many applications such as modeling the distributions of mechanical strength of materials [17,20,21,56], earthquake interevent times [1,2,6,7,8,57,58,59,60,61], and precipitation amounts [62,63,64].

The κ-Weibull distribution is a deformation of the Weibull model introduced in [39,40] to model the distribution of income in economy. The κ-Weibull has a power-law right tail which captures the observed Pareto law followed by income distributions. The κ-Weibull distribution was later applied to model the mechanical strength of materials and earthquake interevent times [43,44].

The κ-Weibull model admits explicit expressions for the main probability functions which are given by the following expressions [44]:

(25a) $$F_{\kappa }\left(x\right)=1-\exp_{\kappa }\left(-{\left[x/x_{s}\right]}^{m}\right)\;,$$

(25b) $$S_{\kappa }\left(x\right)=\exp_{\kappa }\left(-{\left[x/x_{s}\right]}^{m}\right)\;,$$

(25c) $$f_{\kappa }\left(x\right)=\frac{m}{x_{s}}{\left(\frac{x}{x_{s}}\right)}^{m-1}\frac{\exp_{\kappa }\left(-{\left[x/x_{s}\right]}^{m}\right)}{\sqrt{1+\kappa^{2}{\left(x/x_{s}\right)}^{2m}}}\;,$$

(25d) $$h_{\kappa }\left(x\right)=\frac{m}{x_{s}}\frac{{\left(x/x_{s}\right)}^{m-1}}{\sqrt{1+\kappa^{2}{\left(x/x_{s}\right)}^{2m}}}\;,$$

(25e) $$Q_{\kappa }\left(p\right)=\frac{1}{x_{s}}{\left[\ln_{\kappa }\left(\frac{1}{1-p}\right)\right]}^{1/m}\;.$$

Note that due to the asymptotic behavior of the κ-exponential given by (14), the survival function $S_{\kappa }\left(x\right)$ of the κ-Weibull follows a power law with tail exponent −m/κ:

(26) Sκ(x)x→∞∼2κ(x/xs)−m/κ.

**Figure 5:**
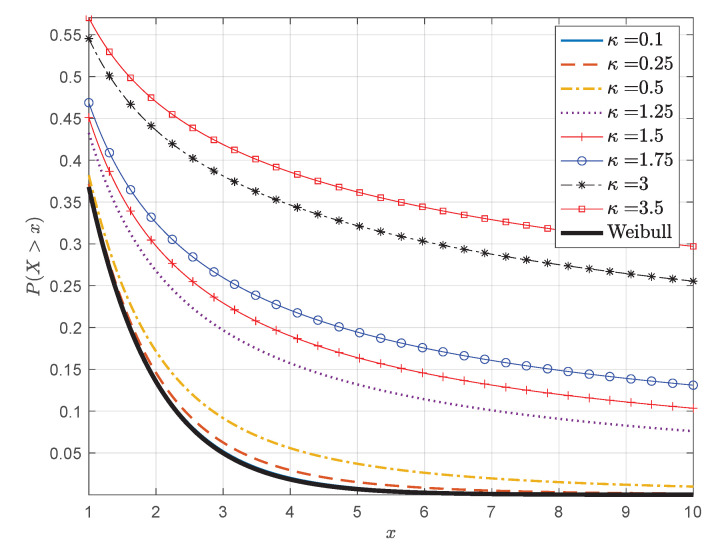
Survival functions for the Weibull and κ-Weibull distributions for different values of κ and $x_{s}=m=1$.

The power-law tail gives the κ-Weibull an advantage over the classical Weibull distribution for systems with algebraic decay of the right tail of the distribution. Figure 5 compares the tails of the survival functions for the Weibull and κ-Weibull models. Note that $S_{\kappa }\left(x\right)$ for κ=0.1 is practically indistinguishable from the Weibull survival function.

### 4.2. Connection with Weakest-Link Scaling Theory

Weakest-link scaling theory underlies the classical Weibull distribution. Weakest-link scaling was proposed by Gumbel [65] and Weibull [66] in their works on the statistics of extreme values. This section provides (i) a brief review of weakest-link scaling in connection with the Weibull model and (ii) a demonstration that the κ-Weibull is also based on the same principle.

Weakest-link scaling treats a disordered system as a chain of critical clusters, also known as links or representative volume elements (RVEs). The term “weakest-link scaling” emphasizes the idea that the strength of an entire system is determined by the strength of its weakest link [67]. The concept of links implies the presence of critical subsystems. The failure of one such subsystem is presumably sufficient to cause failure of the entire system [21]. Thus, weakest-link scaling implies that the survival probability of the system is equal to the product of the link survival probabilities, i.e.,

(27) $$S_{N_{\mathrm{eff}}}\left(x\right)=\prod_{i=1}^{N_{\mathrm{eff}}}S_{1}^{\left(i\right)}\left(x\right),$$

where $N_{\mathrm{eff}}$ is the number of links, $S_{N_{\mathrm{eff}}}\left(x\right)$ is the system survival function, and $S_{1}^{\left(i\right)}\left(x\right)$ is the survival function of the *i*-th link, where $i=1,\ldots ,N_{\mathrm{eff}}$. The above dependence of the survival function is characteristic of brittle fibers and ceramic materials [19,20,22] and justifies the use of the Weibull strength distribution. It has also been shown that if the strength of the earth’s crust follows the Weibull distribution, then the latter is also justified for the distribution of recurrence times between earthquakes under the conditions specified in [18].

Assuming a uniform link survival function $S_{1}\left(x\right)$, the system’s survival function, Equation (27), becomes

(28) $$S_{N_{\mathrm{eff}}}\left(x\right)={\left[S_{1}\left(x\right)\right]}^{N_{\mathrm{eff}}}.$$

The number of links can also be expressed as the ratio of the system’s volume, *V* over the link volume, $V_{0}$, i.e., $N_{\mathrm{eff}}=V/V_{0}$.

To obtain the classical Weibull distribution, the link’s survival function is assumed to have the exponential form $S_{1}\left(x\right)=\exp\left[-{\left(x/x_{l}\right)}^{m}\right]$, where $x_{l}$ is the link’s scale parameter and m>0 is the Weibull modulus or shape parameter [66]. This leads to the system survival function $S_{N_{\mathrm{eff}}}\left(x\right)=\exp\left[-{\left(x/x_{s}\right)}^{m}\right]$, where $x_{s}=x_{l}/N_{\mathrm{eff}}^{1/m}$ is the scale parameter for the entire system.

The κ-Weibull distribution can be obtained by simply replacing the exponential in the Weibull survival function with the deformed κ-exponential. However, this mathematically valid operation does not provide physical motivation for the κ-Weibull. The latter emerges in the framework of weakest-link scaling by using a modified link survival function. More precisely, let us assume that the link survival function depends on the parameter κ as shown in [43,44], i.e., that it satisfies

(29) $$S_{1}\left(x\right)=\sqrt{1+{\left(\frac{x^{m}}{{\tilde{x}}_{l}^{m}}\right)}^{2}}-{\left(\frac{x}{{\tilde{x}}_{l}}\right)}^{m}\;,$$

where ${\tilde{x}}_{l}^{m}=x_{l}^{m}/\kappa$. The parameter κ can be viewed as the inverse number of effective links, that is, $N_{\mathrm{eff}}=1/\kappa$. The link survival $S_{1}\left(x\right)$ thus depends on the number of links, which implies a degree of interactivity in the system. In addition, the asymptotic behavior of $S_{1}\left(x\right)$ for x→∞ is given by a power law, i.e., $S_{1}\left(x\right)∼N_{\mathrm{eff}}\;{\left(2x/x_{l}\right)}^{-m}$.

**Figure 6:**
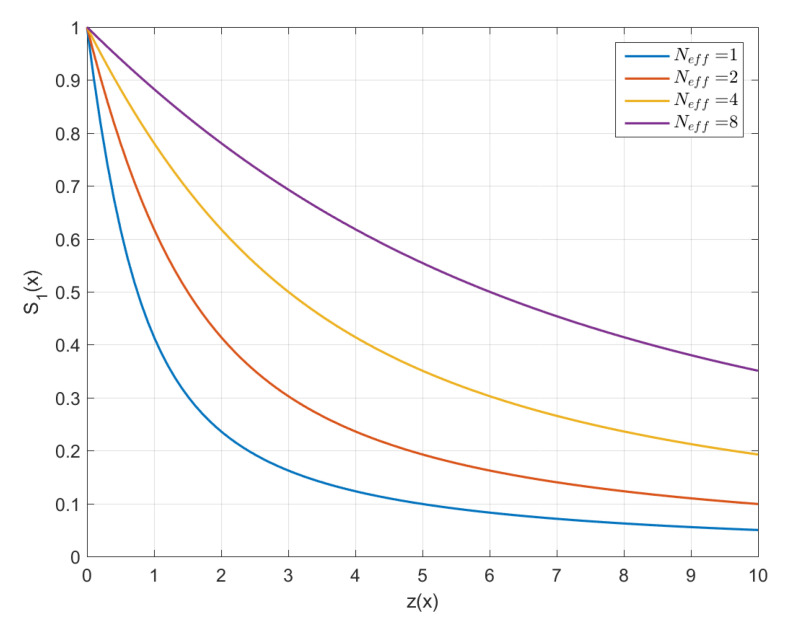
Link survival function for different effective system sizes. The horizontal axis denotes the variable $z\left(x\right)=x^{m}/{\tilde{x}}_{l}^{m}$. Larger values of $N_{\mathrm{eff}}$ correspond to slower decay of $S_{1}\left(x\right)$.

The link survival function for different system sizes $N_{\mathrm{eff}}$ is plotted in Figure 6 against the variable $z\left(x\right)=x^{m}/{\tilde{x}}_{l}^{m}$. The graphs exhibit the power-law asymptotic decline $S_{1}\left(x\right)∼1/z\left(x\right)$ as well as slower decrease of $S_{1}\left(x\right)$ for increasing $N_{\mathrm{eff}}$.

Finally, the weakest-link scaling relation (28) in view of the link survival function (29) and the κ-exponential definition (2) leads to an $S_{N_{\mathrm{eff}}}\left(x\right)$ which is given by the κ survival function (25b).

### 4.3. κ-Weibull Plot for Graphical Testing

If we define the function $\Phi_{\kappa }\left(x\right)=\ln\ln_{\kappa }\left(1/S_{\kappa }\left(x\right)\right)$, it follows from Equation (25b) that $\Phi_{\kappa }\left(x\right)=m\ln\left(x/x_{s}\right)$. This relation suggests a graphical approach to test if a given dataset ${\left\{x_{n}\right\}}_{n=1}^{N}$ follows the κ-Weibull distribution: it suffices to test if the scatter plot of $\Phi_{\kappa }\left(x_{n}\right)$ versus $\ln x_{n}$ (for n=1,…,N) is concentrated around a straight line with slope equal to *m*. This property allows a quick visual test of the fit between the data and the κ-Weibull distribution which is analogous to the widely used Weibull plot [68].

**Figure 7:**
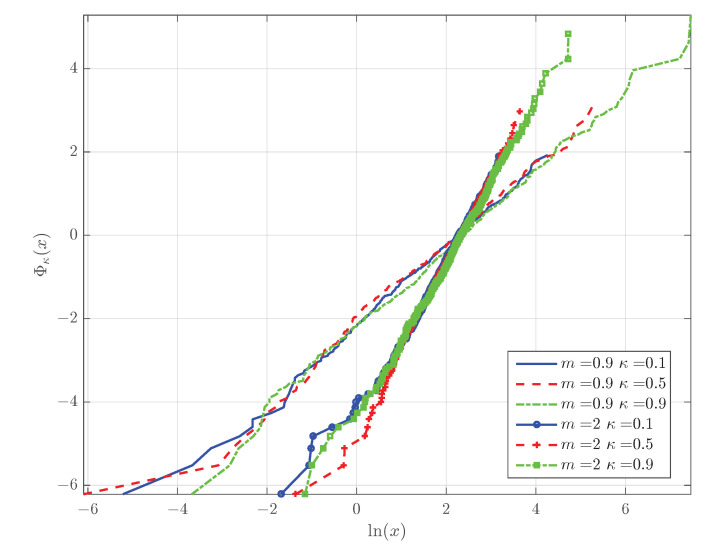
Plots of estimated $\Phi_{\kappa }\left(x\right)$ obtained from κ-Weibull synthetically generated samples with different values of the Weibull modulus *m* and the deformation parameter κ.

The linear dependence of $\Phi_{\kappa }\left(x\right)$ on lnx is illustrated in the κ-Weibull plots of Figure 7. The graphs represent estimates of $\Phi_{\kappa }\left(x\right)$ derived from six samples of 500 random numbers; the latter are generated from the κ-Weibull distribution by using the inverse transform sampling method [43]. The samples are drawn from κ-Weibull distributions with $x_{s}=10$, m=0.9 and with $x_{s}=10$, m=2; the samples with the same *m* value differ with respect to κ which takes values in {0.1,0.5,0.9}. The function $\Phi_{\kappa }\left(x\right)$ is estimated by means of ${\hat{\Phi }}_{\hat{\kappa }}\left(x\right)=\ln\ln_{\hat{\kappa }}\left[1/\hat{S}\left(x\right)\right]$, where $\hat{S}\left(x\right)$ is the estimated survival function using the empirical staircase estimate of the CDF from the data, and $\hat{\kappa }$ is the maximum likelihood estimate of κ.

Although the use of graphical tools for estimating the tail exponent has an intuitive appeal, these tools can also be misleading. Thus, in the next section maximum likelihood estimation is used to determine the κ-Weibull parameters including the tail exponent. Statistical testing methods (e.g., Kolmororov–Smirnov test) can also be used to validate the hypothesis of a particular probability distribution model. Note that if the distribution parameters are not known a priori, but instead are estimated from the data (as is typically done in practice), Kolmororov–Smirnov testing must be implemented by using a Monte Carlo resampling approach as described in [27]. This testing approach was applied to probability models for earthquake recurrence times in [43].

### 4.4. Application to Real Data

We investigate the κ-Weibull as an alternative to the Weibull distribution for different data. These include a dataset comprising measurements of tensile strength of carbon fibres [69], daily averaged wind speed in Cairo (Egypt) [70], thickness of magmatic sheet intrusions (dykes) for different tectonic settings [71], tensile strength of steel [72], and earthquake recurrence times [73,74].

The aforementioned datasets are fitted to the Weibull and κ-Weibull distributions by using maximum likelihood estimation. The Matlab code used to estimate the model parameters is publicly available [75]. The results of the fits are presented in Table 1. The entries include the maximum likelihood estimates of the model parameters as well as the optimal negative log-likelihood (NLL) values for each fit. The lower the NLL of a given distribution model is, the better its fit to a particular dataset. For all the cases listed in Table 1, the NLL is lower for the κ-Weibull than for the Weibull distribution. However, the κ-Weibull involves three parameters whereas the Weibull model involves only two parameters. To account for the different number of parameters, the selection of the optimal model can be performed by means of the Akaike information criterion (AIC) [76], i.e., AIC=2k+2NLL, where *k* is the number of free parameters for each model (k=2 for the Weibull and k=3 for the κ-Weibull). The best model has the lowest AIC value. Because ${\mathrm{AIC}}_{\kappa W}-{\mathrm{AIC}}_{W}=2\left(1+{\mathrm{NLL}}_{\kappa W}-{\mathrm{NLL}}_{W}\right)$, AIC favors the κ-Weibull only if ${\mathrm{NLL}}_{\kappa W}-{\mathrm{NLL}}_{W}<-1$. This condition is satisfied for all but the first two datasets.

A more stringent condition is provided by the Bayesian information criterion, BIC=klogN+2NLL, where *N* is the data size [77]. For N>8, the BIC imposes a bigger penalty on model complexity than AIC. The difference in BIC values for the κ-Weibull and the Weibull models is given by ${\mathrm{BIC}}_{\kappa W}-{\mathrm{BIC}}_{W}=\log N+2\left({\mathrm{NLL}}_{\kappa W}-{\mathrm{NLL}}_{W}\right)$. Thus, under the BIC the κ-Weibull is optimal if ${\mathrm{NLL}}_{\kappa W}-{\mathrm{NLL}}_{W}<-\left(\log N\right)/2$. The BIC condition also favors the κ-Weibull distribution for the datasets 3–9. In order to allow easier comparison, the values of AIC and BIC (divided by the number of sample points) for both the Weibull and κ-Weibull distributions are listed for each dataset in Table 2.

## 5. The κ-Lognormal Distribution

The lognormal distribution is often used to model long-tailed processes [23]. In this section we derive a generalization of the lognormal which is based on the κ-deformation of the exponential function. Let us assume that the random variable *Y* follows the normal distribution with marginal PDF given by

(30) $$f_{y}\left(y\right)=\frac{1}{\sqrt{2\pi }\sigma }\;e^{-{\left(y-m\right)}^{2}/2\sigma^{2}}.$$

We then define the random variable $X=g_{\kappa }\left(Y\right)$ by means of the κ-exponential transformation

(31) $$x=g_{\kappa }\left(y\right)≜\exp_{\kappa }\left(y\right)={\left(\sqrt{1+\kappa^{2}y^{2}}+\kappa y\right)}^{1/\kappa }.$$

To determine the PDF of the random variable *X* we use the standard integral relation for the PDF under the nonlinear transformation $g_{\kappa }\left(y\right)$

$$f_{x}\left(x;\kappa \right)=\int_{-\infty }^{\infty }\delta \left(x-g_{\kappa }\left(y\right)\right)\;f_{y}\left(y\right)\;dy\;.$$

Because the nonlinear transform is monotonic and therefore invertible, the PDF of *X* is given by means of the following mapping [47,52]:

$$f_{x}\left(x;\kappa \right)=f_{y}\left(y=g_{\kappa }^{-1}\left(x\right)\right)\;\left|\frac{dg_{\kappa }^{-1}\left(x\right)}{\;dx}\right|.$$

Taking into account the normal PDF of Equation (30), the inverse transform which is given by $g_{\kappa }^{-1}\left(x\right)=\ln_{\kappa }\left(x\right)$, and the first derivative of $\ln_{\kappa }\left(\cdot \right)$ which is given by Equation (Section 2.2), it follows that the PDF of the κ-lognormal distribution is given by

(32) $$f_{x}\left(x;\kappa \right)=\frac{1}{\sqrt{2\pi }\sigma \;x}\;e^{-{\left(\ln_{\kappa }x-m\right)}^{2}/2\sigma^{2}}\;\left(\frac{x^{\kappa }+x^{-\kappa }}{2}\right)\;.$$

It is clear that $f_{x}\left(x;\kappa =0\right)$ recovers the lognormal distribution because $\lim_{\kappa \to 0}\ln_{\kappa }\left(x\right)=\ln x$ and $\lim_{\kappa \to 0}\left(\frac{x^{\kappa }+x^{-\kappa }}{2}\right)=1$. Moreover, the κ-lognormal PDF given by Equation (32) is symmetric with respect to κ.

The Box–Cox transform of the Gaussian PDF can be similarly obtained by means of the PDF transformation under a change of variables. The resulting PDF is given by

(33) $$f_{x}\left(x;\lambda \right)=\frac{x^{\lambda -1}}{\sqrt{2\pi }\sigma }\;e^{-{\left[(x^{\lambda }-1)/\lambda -m\right]}^{2}/2\sigma^{2}}\;.$$

**Figure 8:**
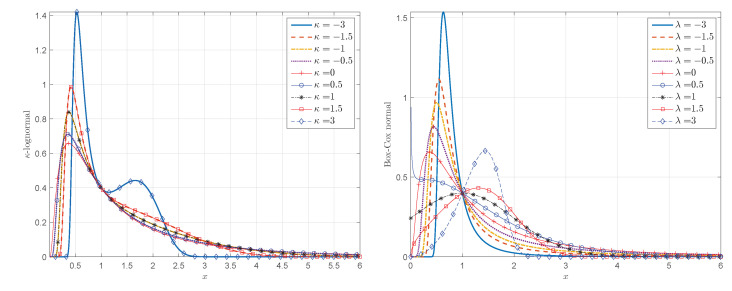
Probability density functions resulting from the κ-logarithmic (**left**) and Box–Cox (**right**) transformations of the standard normal distribution, given by Equations (32) and (33) respectively. The curves correspond to different values of λ=κ (λ is the Box–Cox parameter and κ is the deformation parameter of the Kaniadakis logarithm).

The κ-lognormal PDF given by Equation (32) has heavier tails than the PDF in Equation (33) resulting from the Box–Cox transform of the Gaussian. This is confirmed by the parametric plots of the two parametric PDF families shown in Figure 8 for different values of λ=κ. Notice that for λ=0 (left frame in Figure 8), and κ=0 (right frame in Figure 8) the PDFs tend to the lognormal PDF.

In order to compare the tails of the κ-lognormal with the tails of the lognormal distribution, let us define the PDF ratio $R_{f}\left(x;\kappa \right)$:

(34) $$R_{f}\left(x;\kappa \right)≜\frac{f_{x}\left(x;\kappa \right)}{f_{x}\left(x;\kappa =0\right)}=e^{-\left\{{\left(\ln_{\kappa }x-m\right)}^{2}-{\left(\ln x-m\right)}^{2}\right\}/2\sigma^{2}}\;\left(\frac{x^{\kappa }+x^{-\kappa }}{2}\right)\;.$$

First, we show that for κ>0 and for x>1 it holds that $\ln_{\kappa }\left(x\right)>\ln x$. Let us define the function $u\left(x\right)=\ln_{\kappa }\left(x\right)-\ln x$. (i) It holds that $u\left(1\right)=\ln_{\kappa }\left(1\right)-\ln1=0$. It suffices to show that u(x) is a monotonically increasing function for x>1. (ii) The derivative of u(x) is $du\left(x\right)/dx=\left(x^{\kappa }+x^{-\kappa }-2\right)/\left(2x\right)$, and it is positive if $x^{\kappa }+x^{-\kappa }>2$. By multiplying both sides with $x^{\kappa }$ ($x^{\kappa }>0$) and setting $x^{\kappa }=y$, the inequality $x^{\kappa }+x^{-\kappa }>2$ becomes equivalent to ${\left(y-1\right)}^{2}>0$, which is true for any y∈R. Hence, it holds that $\lim_{x\to \infty }R_{f}\left(x;\kappa \right)=0$, and thus the κ-lognormal has a lighter right tail than the lognormal distribution.

**Figure 9:**
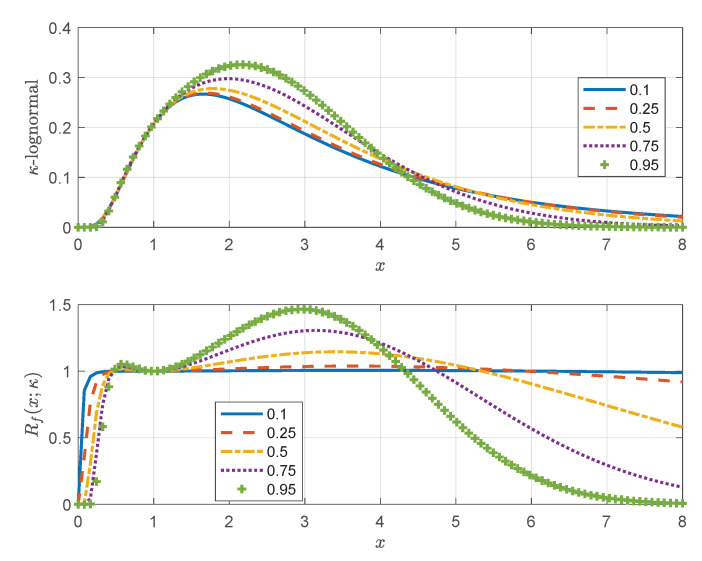
Parametric plots (versus *x*) of the κ-lognormal PDF defined in Equation (32) (**top**) and the ratio function $R_{f}\left(x;\kappa \right)$ defined in Equation (34); the latter compares the tails of the κ-lognormal relative to the lognormal distribution for different values of the deformation parameter κ (**bottom**).

The proof of monotonicity of u(x) also holds for x<1: by replacing *x* with $x^{\prime }=1/x$ where $x^{\prime }>1$, it holds that $x^{\kappa }+x^{-\kappa }={\left(x^{\prime }\right)}^{-\kappa }+{\left(x^{\prime }\right)}^{\kappa }$, and therefore du(x)/dx>0; that is $\ln_{\kappa }\left(x\right)>\ln x$ for x<1. Therefore, $\lim_{x\to 0}R_{f}\left(x;\kappa \right)=0$, and thus the left tail of the κ-lognormal is also lighter than the lognormal’s tail. Hence, the κ-lognormal concentrates more density in the middle of the distribution than the lognormal. The relative transfer of density from the tails to the body of the distribution is controlled by κ and becomes more pronounced as κ increases. This is confirmed by the parametric plots of $R_{f}\left(x;\kappa \right)$ shown in Figure 9 (bottom panel) for different values of κ.

### 5.1. Effective Permeability of Random Media

Single-phase, incompressible, steady-state flow in saturated random media is governed by the partial differential equation

(35) ∇·K(s)∇P(s)=0,with suitable boundary conditions,

where $s\in R^{d}$ is the position vector, P(s) is the pressure, and ∇P(s) the pressure gradient, a·b denotes the inner product of the vectors a and b, and K(s) is the fluid permeability. The latter is assumed to be a scalar random field, i.e., a random function over the domain $D\subset R^{d}$; (in the case of anisotropic media *K* becomes a tensor). Equation (35) is the continuity equation which expresses the conservation of mass.

In the case of slow viscous flow through random media, the fluid velocity is given by Darcy’s law, i.e., V(s)=−K(s)∇P(s)/μ where μ is the fluid viscosity [78], and thus the continuity equation becomes ∇·V(s)=0. The local variations of the velocity do not usually matter for the macroscopic flow behavior, i.e., for the average fluid velocity through a large domain. The macroscopic velocity is often determined in terms of an effective permeability; the latter connects the average pressure gradient to the average fluid velocity by means of $E\left[V\left(s\right)\right]=-K_{eff}E\left[\nabla P\left(s\right)\right]$. The ensemble average here is evaluated over the joint probability distribution of K(s), which represents the local variations (microstructure) of the medium. Similarly, effective measures can be defined for other properties (e.g., elasticity) of porous media by means of averages over the microstructural disorder [79,80,81].

The generalized mean ${\langle K\rangle }_{\alpha }$ with α=1−2/d, also known as the power-law mean, is used to estimate the effective flow permeability (for single-phase, saturated flow), $K_{eff}$ of random porous media with lognormal disorder and short-range correlations [16]. For a given power-law exponent α, the generalized mean is defined by means of the following expectation (assuming that it is well-defined mathematically):

(36) $${\langle K\rangle }_{\alpha }≜{\left\{E[K^{\alpha }]\right\}}^{1/\alpha },\;-1\leq \alpha \leq 1.$$

For α=−1, Equation (36) yields the harmonic mean, whereas for α=0 the geometric mean, $K_{G}=\exp\left[E\left(\ln K\right)\right]$, is obtained as the limit $\lim_{\alpha \to 0}{\langle K\rangle }_{\alpha }$.

Furthermore, assuming that K=g(Y) where $Y∼\left(m,\sigma^{2}\right)$ is a normally distributed random variable. In general, both *Y* and *K* can be random fields with spatial correlations. However, this does not affect the calculation of the generalized mean, which is a point (marginal) property. The generalized mean of K=g(Y) is now given by

(37) $${\langle K\rangle }_{\alpha }≜{\left\{E_{Y}\left[g^{\alpha }\left(Y\right)\right]\right\}}^{1/\alpha },\;-1\leq \alpha \leq 1.$$

Thus, if *K* follows the lognormal distribution, namely, K=exp(Y), it holds that

(38) $${\langle K\rangle }_{\alpha }={\left\{E_{Y}\left[\exp\left(\alpha Y\right)\right]\right\}}^{1/\alpha }=e^{m+\frac{\alpha }{2}\;\sigma^{2}},\;-1\leq \alpha \leq 1.$$

This equation, known as the Landau–Lifshitz–Matheron (LLM) ansatz, was first proposed for the dielectric permittivity of random dielectric mixtures [82]. In the case of electromagnetism, the continuity equation is embodied in Gauss’s law; for zero free charge density the latter becomes ∇·D(s)=0, where D(s)=−ϵ(s)∇ϕ(s) is the dielectric displacement field, ϕ(s) is the applied electric potential, and ϵ(s) is the dielectric permittivity of the medium. Notation differences aside, the mathematical form of the electrostatic equations is identical to those of the fluid flow problem; in both cases the continuity principle results in an elliptic partial differential equation with suitable boundary conditions. This reason underlies LLM’s applicability to both fluid permeability and dielectric permittivity; as Richard Feynman wrote ([83], Chap. 12.1): “there is a most remarkable coincidence: The equations for many different physical situations have exactly the same appearance”.

For d=1 (e.g., for pipe flows), LLM yields the harmonic mean $K_{H}=1/E\left[K^{-1}\right]$; in this case the flow is cut off if the permeability vanishes at a single point. For d=2, $K_{eff}={\langle K\rangle }_{0}$ yields the geometric mean, $K_{G}=\exp\left(E\left[\ln K\right]\right)$, which coincides with the exact solution in two dimensions [84]. Finally, in d=3 the expression ${\langle K\rangle }_{1/3}$ follows from perturbative renormalization group analysis [16]. The LLM equation implies that the effect of disorder (as measured by the log-permeability variance $\sigma^{2}$) is reduced as the embedding dimension of the medium increases. The physical meaning of this dependence is that three-dimensional media include more permeable paths than one- or two-dimensional random media, thus enabling the bypass of flow bottlenecks.

### 5.2. Generalized Mean of the κ-Lognormal Distribution

This section focuses on the calculation of the generalized mean when *K* follows the κ-lognormal distribution. In this case, the respective ensemble average over the normal variable $Y∼\left(m,\sigma^{2}\right)$, defined by Equation (37), is given by

(39) $${\langle K\rangle }_{\alpha ;\kappa }={\left\{E_{Y}\left[{\left\{\exp_{\kappa }\left(Y\right)\right\}}^{\alpha }\right]\right\}}^{1/\alpha }={\left\{E_{Y}\left[\exp_{\kappa /\alpha }\left(\alpha Y\right)\right]\right\}}^{1/\alpha },\;-1\leq \alpha \leq 1,$$

where in deriving the last equality above we used the identity

(40) $${\left[\exp_{\kappa }\left(Y\right)\right]}^{\alpha }=\exp_{\kappa /\alpha }\left(\alpha Y\right)=\exp_{\kappa^{\prime }}\left(\alpha Y\right),\;where\;\kappa^{\prime }=\kappa /\alpha .$$

Note that for κ=0 Equation (39) is equivalent to Equation (38) because the κ-exponential is reduced to the standard exponential.

If, on the other hand, κ>0, it holds that $\kappa^{\prime }\neq 0$. The expectation in Equation (39) can then be calculated by using the Taylor series expansion of $\exp_{\kappa /\alpha }\left(\alpha Y\right)$ around exp(αY); according to Equation (4), the expansion is given by

(41) $$\exp_{\kappa^{\prime }}\left(\alpha y\right)=\exp\left(\alpha y\right)+\sum_{n=0}^{\infty }\left[\xi_{n}\left(\kappa^{\prime }\right)-1\right]\;\frac{{\left(\alpha y\right)}^{n}}{n!},\;\mathrm{where}\;\kappa^{\prime }=\kappa /\alpha \in R,$$

and the polynomials $\xi_{n}\left(\cdot \right)$ are defined in Equation (5).

The power series in Equation (41) represents the correction of $\exp_{\kappa^{\prime }}\left(\alpha y\right)$ with respect to exp(αy). Based on Equation (7), it holds that $\exp_{\kappa^{\prime }}\left(\alpha y\right)=\exp\left(\alpha y\right)+O\left({\kappa^{\prime }}^{2}\right)$. Notice that $\xi_{n}\left(\kappa^{\prime }\right)-1=0$ for n=0,1,2, whereas $\xi_{n}\left(\kappa^{\prime }\right)-1<0$ for n=3,4. Hence,

(42) $$\exp_{\kappa^{\prime }}\left(\alpha y\right)=\exp\left(\alpha y\right)+\sum_{n=3}^{\infty }\left[\;\xi_{n}\left(\kappa^{\prime }\right)-1\right]\;\frac{{\left(\alpha y\right)}^{n}}{n!}.$$

Hence, the expectation of the above is given by means of the following expression:

(43) $$E_{Y}\left[\exp_{\kappa^{\prime }}\left(\alpha Y\right)\right]=E_{Y}\left[\exp\left(\alpha Y\right)\right]+\sum_{n=3}^{\infty }\left[\;\xi_{n}\left(\kappa^{\prime }\right)-1\right]\;\frac{\alpha^{n}}{n!}\;E_{Y}\left[Y^{n}\right],\;\kappa^{\prime }=\kappa /\alpha .$$

The expectation $E_{Y}\left[Y^{n}\right]$ is given by means of the following expression by using the fluctuation $Y^{\prime }=Y-m$ and Newton’s binomial formula:

EYYn=EY(m+Y′)n=∑j=0nnjmn−jEY′Y′j.

The expectation over the fluctuations can be calculated by using the Wick–Isserlis theorem [52] $E_{Y^{\prime }}\left[Y^{2\ell }\right]=\left(2\ell \right)!\;\sigma^{2\ell }/\left(2^{\ell }\ell !\right)$, and $E_{Y}\left[Y^{2\ell +1}\right]=0$ for ℓ∈N.

Therefore the difference between the generalized mean of the κ-lognormal and the generalized mean of the lognormal (for the same value of α) is given by an infinite power series (correction factor), where $\delta_{j,2\ell }$ is the Kronecker delta, as follows:

(44) $$E_{Y}\left[\exp_{\kappa^{\prime }}\left(\alpha Y\right)\right]=E_{Y}\left[\exp\left(\alpha Y\right)\right]+\sum_{n=3}^{\infty }\alpha^{n}\left[\xi_{n}\left(\kappa^{\prime }\right)-1\right]\;\sum_{j=0}^{n}m^{n-j}\;\frac{\sigma^{j}\;\delta_{j,2\ell }}{2^{j/2}\left(n-j\right)!\left(j/2\right)!}.$$

Finally, returning to Equation (39) and using Equation (44) the generalized mean of the κ-lognormal distribution is given by

(45) $${\langle K\rangle }_{\alpha ;\kappa }={\langle K\rangle }_{\alpha }\;{\left[1+\frac{\sum_{n=3}^{\infty }\sum_{j=0}^{n}\alpha^{n}\left[\xi_{n}\left(\kappa /\alpha \right)-1\right]\;\frac{m^{n-j}\;\sigma^{j}\;\delta_{j,2\ell }}{2^{j/2}\left(n-j\right)!\;\left(j/2\right)!}}{{\langle K\rangle }_{\alpha }^{\alpha }}\right]}^{1/\alpha }\;.$$

The convergence of the power series in Equation (45) should be further investigated mathematically. To gain some insight into Equation (45), consider the case α=1, which corresponds to the arithmetic (linear) mean. Then, the arithmetic mean of the κ-lognormal, i.e., ${\bar{K}}_{\kappa }≜{\langle K\rangle }_{\alpha =1;\kappa }$ is given by

(46) $${\bar{K}}_{\kappa }=\bar{K}\left[1+\frac{1}{\bar{K}}\sum_{n=3}^{\infty }\sum_{j=0}^{n}\left[\xi_{n}\left(\kappa \right)-1\right]\;\delta_{j,2\ell }\;\frac{m^{n-j}\;\sigma^{j}\;}{2^{j/2}\left(n-j\right)!\;\left(j/2\right)!}\right]\;.$$

Hence, the arithmetic mean of the κ-lognormal is given by the standard arithmetic mean plus a correction factor which involves a double power series. Notice that when κ=0, according to Equation (5) it holds $\xi_{n}\left(\kappa =0\right)=1$ for all n∈N; hence, Equation (46) recovers the arithmetic mean when κ=0 because the double power series vanishes.

**Figure 10:**
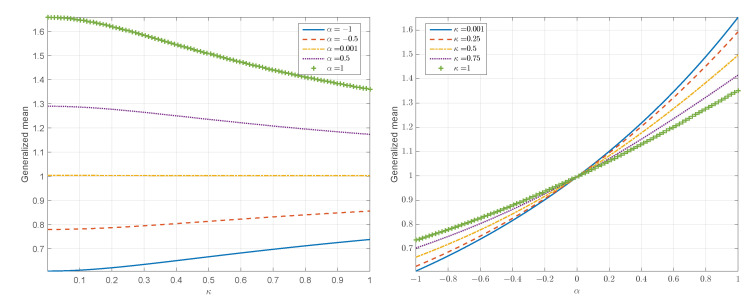
Parametric plots of the generalized mean versus κ for different values of the averaging exponent α (**left**) and the generalized mean versus α for different values of the deformation parameter κ (**right**).

On a more practical note, a numerical calculation of the generalized mean shows that the infinite series in ${\langle K\rangle }_{\alpha ;\kappa }$ converges for −1≤α≤1 and 0<κ<1. Figure 10 shows parametric plots of the generalized mean obtained by a numerical evaluation of Equation (39). The expectations (for different α and κ) are calculated by using an ensemble of $10^{4}$ random variates drawn from the standard normal distribution, i.e., Y∼N(0,1).

The plots on the left of Figure 10 display the generalized mean as a function of κ for different values of the averaging exponent α. As evidenced in these plots, the difference between the harmonic mean (α=−1) and the arithmetic mean (α=1) is reduced as κ increases. This behavior is due to the smaller tail weight of the κ-lognormal PDF for κ↑. It is also observed that the geometric mean (α=0) is independent of κ (for numerical reasons we use α=0.001 instead of α=0). This is also understood in light of Equation (39). Finally, the arithmetic mean (more generally, the generalized mean for α>0) decays with increasing κ whereas the harmonic mean (more generally, the generalized mean for α<0) increases. This behavior is due to the fact that the arithmetic mean reflects the diminishing right tail of the κ-lognormal as κ increases. On the other hand, the harmonic mean is more strongly influenced by the lower part of the distribution; according to Figure 9 the κ-lognormal has higher density in the left tail (except for values very close to zero) for higher values of κ.

The plots on the right of Figure 10 display the generalized mean as a function of α for different values of κ. All the curves exhibit an increase of the generalized mean with increasing α. This reflects the progression from the harmonic to the arithmetic mean according to the well-known ordering $K_{H}\leq K_{G}\leq \bar{K}$. All the curves intersect at α=0, marking the independence of the generalized geometric mean on κ. Finally, the slope of the curves is reduced with increasing κ due to the respective shrinking of the tails of the κ-lognormal.

## 6. Discussion

Section 3 introduced and investigated the properties of a nonlinear normalizing transform which is based on the κ-logarithm. The proposal generalizes the Box–Cox transform, and it can be used for normalizing skewed data before geostatistical methods are applied. An application to precipitation time series modeling was presented. Note that the nonlinear κ-logarithm transform could also be used in spatial models of precipitation in the framework of the censored latent Gaussian field approach [10]. At fine spatiotemporal scales, the correlations of dry/wet spells as well as storm autocorrelation patterns can be better captured by means of two-state models that use copulas to simulate the dependence structure [85]. We believe that the κ-Weibull and κ-lognormal distributions discussed herein will be useful in the framework of two-state models as well, e.g., for modeling the intensity of wet spells.

Section 4 shows that the κ-Weibull model, which is a deformation of the classical Weibull with a power-law right tail, also respects the principle of weak-link scaling. Various extensions of the Weibull model have been proposed in the scientific literature, e.g., [86,87]. However, some of these models provide deformations of the Weibull expression that fail the weakest scaling equation (see Equation (28)) as pointed out by Zok [22]. Such modifications, although mathematically permissible, lack the physical justification of the classical Weibull model which is based on weakest-link scaling. Moreover, we established that the link statistical properties depend on the number of links in the system. This feature indicates a strongly interacting system; alternatively, it shows that the observed system is a part of a larger system.

The asymmetric κ-lognormal distribution introduced in Section 5 has lighter tails than the lognormal distribution. The deviation from the lognormal is controlled by the parameter κ. Smaller values of κ≈0 imply small deviations, whereas larger values of κ≈1 signify thinner tails than the lognormal. The κ-lognormal can be used as a model of fluid permeability for random porous media. We believe that the stochastic theory of single-phase, saturated fluid flow in κ-lognormal media can be derived, at least in the framework of perturbation analysis, and expressions for the effective permeability can be likewise obtained. An interesting question is how the parameter κ which controls tail behavior will impact flow properties.

We have reviewed the generalized mean for the lognormal distribution and its application in the estimation of the effective permeability of random media. We then studied the generalized mean for the κ-lognormal distribution. Note that our calculations do not prove that the effective permeability of random media with κ-lognormal disorder is given by the generalized mean. This intriguing hypothesis should be further explored in the framework of the stochastic theory of flow and transport [80].

## 7. Conclusions

We presented applications of the Kaniadakis κ-exponential and κ-logarithm functions in the modeling of mechanical strength and in earth science problems. In particular, we focused on κ deformations of classical distribution models such as the Weibull and the lognormal. The κ-Weibull distribution has a power-law tail which is useful for the modeling of mechanical strength, earthquake recurrence times, and properties of geological structures, among other applications. On the other hand, the κ-lognormal model has a tail lighter than the lognormal; this feature is of interest for skewed distributions which decline faster than the lognormal. The methodological applications of the Kaniadakis functions presented include the following:

- The modified Box–Cox transform given by Equation (22).
- Application of the modified Box–Cox transform to an autoregressive, intermittent model of precipitation as described in Section 3.3.
- Connection between the κ-Weibull probability model with the theory of weakest-link scaling as shown in Section 4.2.
- The study of the κ-lognormal distribution which is a generalization of the lognormal model with lighter tails. The PDF of this new model is given by Equation (32).
- The calculation of the power-mean (generalized mean) of the κ-lognormal as shown in Section 5.2.

Further study of probability models based on the deformed exponential and logarithmic functions will lead to significant advances in different fields of earth science. The most obvious applications at this time include (i) modeling the mechanical strength of technological materials and geologic media, earthquake recurrence times, wind speed, and precipitation amounts, (ii) nonlinear transforms used for Gaussian anamorphosis in geostatistical and ensemble Kalman filtering applications [88], and (iii) the permeability of random porous media.

## Figures and Tables

**Table 1:** Results of maximum likelihood estimated fits to the Weibull and κ-Weibull distribution. 1. Tensile strength of carbon fibers. 2. Daily averaged wind speeds from 1 January 2009 to 4 October 2009 for Cairo, Egypt. 3–6. Thickness of magmatic sheet intrusions for different tectonic settings. 7. Tensile strength of low-alloy steels. 8. Recurrence times of aftershocks (A.R.T.) from 25 October 2018 until 31 May 2019, following the major M_w_ 6.9 Zakynthos earthquake (Greece). 9. Recurrence times of foreshocks (F.R.T.) preceding the Zakynthos earthquake (from 1 January 2014 until 25 October 2018). For more information regarding the data see the relevant sources. *N*, sample length; $x_{s}$, scale parameter; *m*, shape parameter; κ, Weibull deformation parameter. Values are rounded off to the second decimal digit. NLL, Negative log-likelihood.

		Weibull			κ-Weibull			
**Data**	N	$x_{s}$	m	**NLL**	$x_{s}$	m	κ	**NLL**
1. C fibers (GPa) [69]	100	2.94	2.79	141.53	2.90	2.98	0.285	141.23
2. Wind (mph) [70]	100	8.05	2.78	240.21	7.63	3.28	0.56	239.32
3. Dyrfjöll (m) [71]	487	0.90	1.26	378.05	0.84	1.46	0.42	368.40
4. Geitafell (m) [71]	546	0.57	1.02	233.88	0.52	1.17	0.43	225.62
5. Tenerife (m) [71]	550	1.83	1.02	875.18	1.65	1.18	0.45	867.38
6. La Palma (m) [71]	2093	0.43	1.14	206.51	0.37	1.53	0.66	83.98
7. Steel (MPa) [72]	915	548.58	1.98	6194.22	524.26	4.81	0.52	5753.13
8. A.R.T. (days) [74]	7822	0.027	0.94	−20207	0.024	1.19	0.49	−20374
9. F.R.T. (days) [74]	4731	0.28	0.68	−692.60	0.27	0.70	0.17	−698.37

**Table 2:** Measures of fit to the Weibull and κ-Weibull distributions for the datasets listed in Table 1. NLL, negative log-likelihood; AIC’, value of Akaike information criterion value per sample point, i.e., AIC’ = AIC/N = 2(k+NLL)/N; BIC’, value of Bayesian information criterion per sample point, i.e., BIC’ = BIC/N = (klogN+2NLL)/N.

		Weibull			κ-Weibull		
**Data**	N	**NLL**	**AIC’**	**BIC’**	**NLL**	**AIC’**	**BIC’**
1. C fibers (GPa)	100	141.53	2.8706	2.9227	141.23	2.8846	2.9628
2. Wind (mph)	100	240.21	4.8842	4.8963	239.32	4.8464	4.9246
3. Dyrfjöll (m)	487	378.05	1.5608	1.5780	368.40	1.5253	1.5511
4. Geitafell (m)	546	233.88	0.8640	0.8798	225.62	0.8374	0.8611
5. Tenerife (m)	550	875.18	3.1897	3.2054	867.38	3.1650	3.1885
6. La Palma (m)	2093	206.51	0.1992	0.2046	83.98	0.0831	0.0912
7. Steel (MPa)	915	6194.22	13.5437	13.5542	5753.13	12.5817	12.5975
8. A.R.T. (days)	7822	−20207	−5.1662	−5.1644	−20374	−5.2086	−5.2060
9. F.R.T. (days)	4731	−692.60	−0.2919	−0.2892	−692.60	−0.2940	−0.2899

## Data Availability

The datasets analyzed herein can be obtained from the respective publications cited in the paper. Matlab software for estimating the optimal κ-Weibull fits to the data is publicly available from [75].

## Abbreviations

The following abbreviations are used in this manuscript:

AR	Autoregressive
AIC	Akaike Information Criterion
BCT	Box-Cox transform
BIC	Bayesian Information Criterion
CDF	Cumulative distribution function
KLT	κ-logarithm transform
LLM	Landau-Lifshitz-Matheron (ansatz)
NLL	Negative log-likelihood
PDF	Probability density function
